# *Serratia marcescens* as a cause of unfavorable outcome in the twin pregnancy

**DOI:** 10.1515/med-2021-0205

**Published:** 2020-12-17

**Authors:** Duško Kljakić, Miloš Z. Milosavljević, Milan Jovanović, Vesna Čolaković Popović, Saša Raičević

**Affiliations:** Department of Gynecology, General Hospital Bar, Bar, Montenegro; Department of Pathology, University Medical Center Kragujevac, Kragujevac, Serbia; Clinic for Infectious Diseases, Clinical Center of Montenegro, Podgorica, Montenegro; Clinic of Gynecology and Obstetrics, Clinical Center of Montenegro, Podgorica, Montenegro; Medical faculty, University of Montenegro, Podgorica, Montenegro

**Keywords:** twins dizygotic, fetal growth retardation, premature birth, infectious pregnancy complications, *Serratia marcescens*

## Abstract

Several *Serratia* species are widely distributed in nature, but *Serratia marcescens* is the only species frequently isolated in hospitals. This pathogen is mainly responsible for nosocomial infection, mostly in immunocompromised hosts. A 26-year-old woman with a twin pregnancy, regularly controlled, was hospitalized at 24 + 5 weeks of gestation due to scant vaginal bleeding, lower abdominal pain, and body temperature up to 37.5°C. Gynecological examination revealed bleeding accompanied by dilatation of the cervix. The laboratory analyses revealed leukocytosis with elevated C-reactive protein (CRP). Treatment was initiated with intravenous antibiotic administration. After admission, fetal membranes spontaneously ruptured, and an extremely preterm dichorionic female twin birth occurred at 25 + 0 weeks of gestation. Both infants died two days after labor. Pathological and microbiological analyses revealed chorioamnionitis caused by *S. marcescens*. According to the antibiogram, antibiotic treatment was continued for the next 7 days. The examination of cervical and vaginal discharge samples was negative three days and two weeks after therapy. *S. marcescens* may cause spontaneous miscarriages and, in this important case, caused loss of discordant twins in an extremely preterm birth by an immunocompetent patient. Infection by *S. marcescens* cannot be excluded as a cause of discordant growth and needs to be confirmed by further research.

## 1 Introduction

*Serratia marcescens* is an aerobic (facultative anaerobic), motile, Gram-negative, enteric saprophytic rod of the *Klebsiella–Enterobacter–Serratia* division of the *Enterobacteriaceae* family [1]. *S. marcescens* is observed almost everywhere in nature, but it favors moist conditions [2]. In this paper, a case of *S. marcescens* placental infection is described as a cause of extremely preterm birth and loss of infants in twin pregnancy due to pneumonitis followed by respiratory insufficiency.

Infections during pregnancy are complex and important because the infection affects not only the mother, but also the fetus [3]. The duration and results of the infection depend on the number and virulence of the microbes, the immunobiological characteristics of the mother, the manner of spread of the infection, and the gestational period [4]. The immunobiological status of the mother is stressed given the depressed T lymphocyte function and frequent compositional alterations in biological flora in the birth canal during pregnancy, which significantly contributes to the development of infections [5].

As a result of infections during pregnancy, fetal membranes may become infected (chorioamnionitis and intraamniotic infection syndrome), resulting in premature placental abruption and spontaneous miscarriage or preterm birth [6]. Due to the very high infant death rate under these conditions, the peripartum morbidity of the mother is also high [7]. In line with these findings, *Serratia* bacteremia has a high mortality rate of approximately 37% within six months [8]. During pregnancy, this infection is a rare but potentially fatal disorder that is also associated with chorioamnionitis or placental abscess, miscarriages, and preterm deliveries [9,10,11,12,13,14].

## 2 Case report

A 26-year-old (gravida 2, para 1), Rh-negative, childbearing mother with an Rh-positive partner undergoing a regularly controlled second dichorionic twin pregnancy was the subject of this study. The first pregnancy proceeded normally and yielded a positive outcome. Due to uterine cervical insufficiency during this pregnancy, a cerclage was placed at 15 weeks of gestation (WG). Discordant fetal growth restriction (FGR) was diagnosed by ultrasound at 24 + 3 WG based on an estimated discordance of approximately 25% (ultrasound parameters of the first twin: 61 mm biparietal diameter (BPD), 45 mm femur length (FL), 19.8 cm abdominal circumference (AC), 22.6 cm head circumference (HC), and approximately 530 g estimated fetal weight (EFW); ultrasound parameters of the 2nd twin: 65 mm BPD, 48 mm FL, 21 cm AC, 23.8 cm HC, and approximately 730 g EFW.

**Figure 1:**
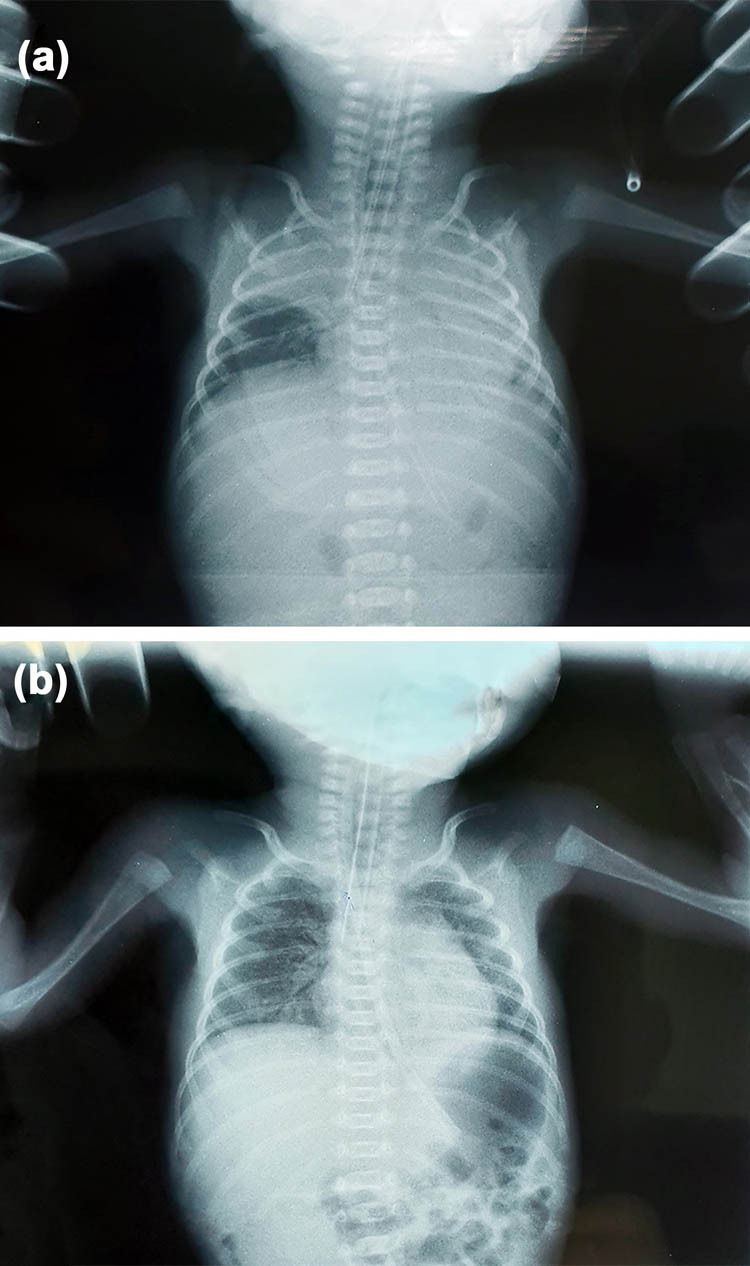
Chest X-ray of the newborns. (a) Image shows decreased transparency of the pulmonary parenchyma to the left with a clearly limited presence in the upper lobe area to the right in the first newborn; (b) chest X-ray shows individual paracardial and basal shadings of the pulmonary parenchyma in the second newborn.

The patient was admitted to the hospital at 24 + 5 WG for scant vaginal bleeding, lower abdominal pain, and body temperature up to 37.5°C. Gynecological examination revealed bleeding within dilatation of the cervix with a diameter of 1 cm near the cerclage. The laboratory analyses revealed leukocytosis with elevated C-reactive protein (CRP) (28.3 × 10^9^/L white blood cells (WBCs), 3.50 × 10^9^/L red blood cells (RBCs), 108 g/L hemoglobin (Hgb), 385 × 10^9^/L platelet count (PLT), and 28.5 mg/L CRP. Other laboratory analyses were in range with reference values. Treatment was initiated with intravenous administration of ceftriaxone at a dose of 2 g/day. Immediately after admission, fetal membranes spontaneously ruptured, and the mother experienced uterine contractions. The cerclage was removed, and an extremely preterm dichorionic female twin birth with discordant FGR occurred at 25 + 0 WG. The first twin weighed 560 g and had an AS of 2/2, and the second twin weighed 780 g and had an AS of 2/2. Both female infants had an altered state of consciousness and were edematous, hypotonic, apneic, and bradycardic, with multiple hematomas at the extremities and in the occipital region, and had leukocytosis (95.6 × 10^9^/L WBCs in the first and 38.9 × 10^9^/L WBCs in the second newborn). Swabs of the umbilical cord, nose, and anus were negative for both infants. Chest X-ray of the first newborn showed decreased transparency of the pulmonary parenchyma to the left with a clearly limited presence in the upper lobe area to the right (Figure 1a). Radiography of the second infant confirmed individual paracardial and basal shadings of the pulmonary parenchyma (Figure 1b). Ultrasound indicated the immaturity of the brain parenchyma in both newborns with asymmetry of the ventricular system and plexus. Both infants were attached to mechanical ventilation, followed by treatment with electrolytes, a prophylactic dose of corticosteroids, and antibiotic therapy. The infants died two days after birth due to respiratory failure despite resuscitation.

**Figure 2:**
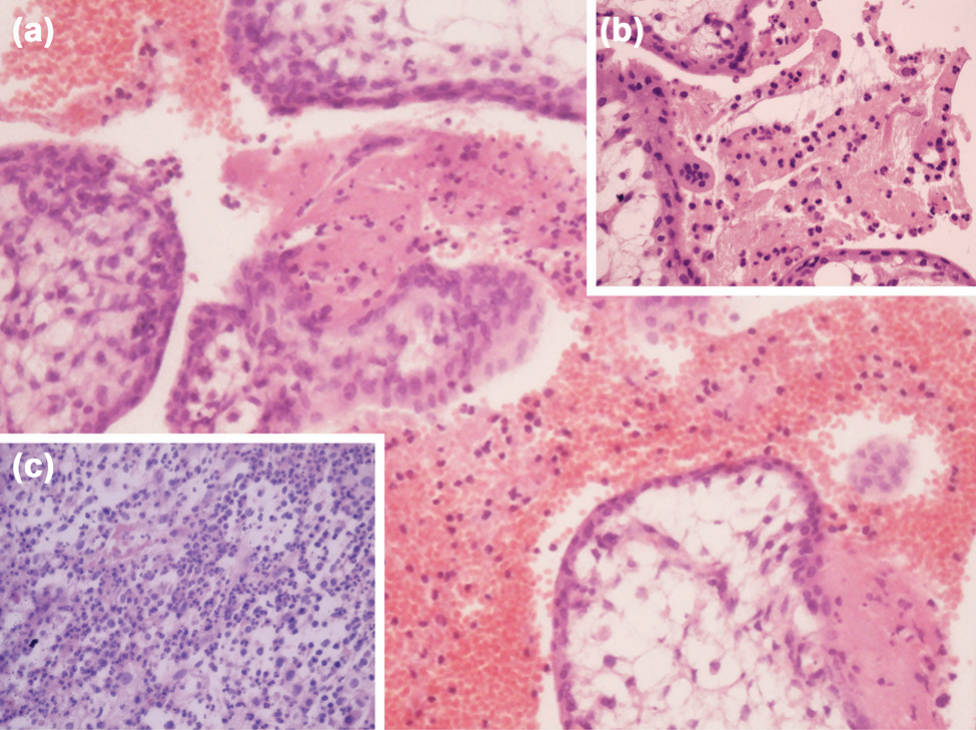
Histological features of chorioamnionitis caused by *Serratia marcescens*. Photomicrographs of the placental tissue show neutrophilic infiltrate of membranes and those overlying the chorionic plate with rare macrophages and without necrotic debris; (a) hematoxylin and eosin staining, ×200; (b) hematoxylin and eosin staining, ×400; (c) hematoxylin and eosin staining, ×400.


The placental tissue was sent for pathological and microbiological analyses. Pathological examination of placental tissue samples showed marginal hemorrhage and opaque membranes with yellow-green discoloration and purulent amniotic fluid. Microscopic analysis showed neutrophilic infiltrate of membranes and those overlying the chorionic plate with rare macrophages and without necrosis, including umbilical vasculitis. A diagnosis of acute chorioamnionitis was made (Figure 2). According to the Amsterdam consensus criteria for maternal and fetal inflammatory responses, our case was classified as Stage 2, Grade 2 [15]. Tests for the presence of genital mycoplasma (*Ureaplasma urealyticum* and *Mycoplasma hominis*) were negative, and real-time polymerase chain reaction (PCR) tests assessing the presence of *Neisseria gonorrhoeae* and *Chlamydia trachomatis* were negative. Applying the same examinations, the presence of the bacteria *S. marcescens* was discovered. Moreover, in a touch preparation of the placental tissue, dead cells were identified (up to 30 leukocytes in the visible range) [16]. After delivery on the third day of hospitalization, treatment was continued with amikacin (1 g/day) and ceftriaxone (2 g/day). On the ninth day of admission, the patient was discharged and treatment was continued according to the antibiogram, with ciprofloxacin at an oral dose of 1 g/day for the next 7 days. Control cervical and vaginal discharge samples were negative three days and two weeks after therapy.

**Informed consent:** Informed consent has been obtained from a mother for potentially descriptive information to be published in this article.

## 3 Discussion

According to the available data, an infection caused by *S. marcescens* results in a miscarriage or extremely preterm birth by an immunocompetent patient, thereby making this case very interesting [13]. Registered in 1960, this bacterial species is often described as a cause of nosocomial interhospital infections [2]. Although this species is sensitive to a wide range of available antibiotics, it is often difficult to exterminate this species from the facilities implicated in interhospital infections [2].

Van Ogtrop et al. followed an outbreak of colonization and infection with *S. marcescens* that occurred in a neonatal intensive care unit [17]. *S. marcescens* was isolated from five preterm infants between 25–30 weeks of gestation. Two infants developed fatal septicemia, and one infant experienced conjunctivitis due to *S. marcescens* [17]. Two infants were colonized, but did not display clinical signs of infection. All infants were treated with antibiotic regimens, including ciprofloxacin and gentamicin [17].

David et al. analyzed twenty-one patients who were infected or colonized in a neonatal unit over a 9-month period from 2001–2002 [18]. Twenty-two isolates were examined for antibiotic susceptibility, β-lactamase production, and genotype [18]. Random-amplified polymorphic deoxyribonucleic acid (DNA) PCR and pulsed-field gel electrophoresis revealed that two clones were present [18]. The first clone caused invasive clinical infection in four babies and was subsequently replaced by a noninvasive clone that affected 14 babies [18]. According to their production of prodigiosin, two different strains have been described: the first strain was nonpigmented, while the second exhibited pink-red pigmentation [18]. The clinical features suggested the difference in the scope of pathogenicities of these two strains. No environmental source was identified [18].

*S. marcescens* has only recently been implicated as a cause of miscarriages and preterm labor resulting from bacteremia and chorioamnionitis [9,10,12]. Prior rupture of membranes is not necessarily due to the development of an ascending amniotic infection [19]. It has been established that subclinical intrauterine infection may occur, even with intact membranes, leading to the absence of clinical signs of infection, despite clear histological signs of chorioamnionitis [20]. The way the infection occurred and spread in this case is not clear. In some cases, the growth and spread of the infection occurred from the vagina, which was confirmed by a vaginal swab [9,13,14]. *S. marcescens* is not part of the normal vaginal flora and is most commonly encountered as an opportunistic pathogen in nosocomial settings [21]. It is typically associated with the use of invasive devices or procedures (e.g., chorionic villus sampling, placement of a central venous line), repeated vaginal examinations after preterm prelabor rupture of membranes as well as with patients whose health is generally compromised [9,11,14]. It is also associated with poor hygiene in health care facilities (hands of personnel, contaminated irrigation solutions or disinfectants) and prior unsuccessful antibiotic treatments of patients [22]. In the hospital, *Serratia* species tend to colonize the respiratory and urinary tracts of adults rather than the gastrointestinal tract (2).

Amniocentesis is very important in the diagnosis of chorioamnionitis. This invasive procedure is followed by risks of miscarriage and transmission of the infection to the fetus [21]. On the other hand, a negative result cannot completely exclude chorioamnionitis, especially at an early stage [11]. In this case, this procedure was not performed. A review of the reported cases of *Serratia marcescens* chorioamnionitis is presented in Table 1.

**Table 1:** Review of the reported cases of *Serratia marcescens* chorioamnionitis

No	Reference	Year	Age	WG	Immunocompetent host	Source of infection	Symptoms on admission	Outcome	Treatment	Length of hospitalization
1.	Kljakic et al. (Current study)	2020	26	25	Yes	Unknown	Scant vaginal bleeding, lower abdominal pain, fever	Vaginal preterm delivery; Infants died two days after birth.	Ceftriaxone (3 days). After delivery, the treatment was continued with Amikacin and Ceftriaxone (6 days) and followed by Ciprofloxacin according to the antibiogram (7 days)	9 days
2.	Mak et al. [21]	2018	35	15–37	Yes	Ascending infection from the vagina	Fever, chills, rigor, runny nose, cough, sputum, headache, myalgia, vomiting undigested food	Vaginal term delivery (uninfected newborn)	Meropenem	24 weeks
3.	Erenberg et al. [14]	2017	36	25–28	Yes	Prolonged PPROM	Chills, abdominal pain, sub-febrile fever, tachycardia, leukocytosis, fetal tachycardia	Emergency cesarean delivery	Amoxicillin/Clavulanic Acid (3 days), followed by Meropenem (6 days)	11 days
4.	Vale-Fernandes et al. [13]	2015	31	15–16	Yes	N/A	Hyperthermia, hemicranial headache, nausea, vomiting, diarrhea	Spontaneous abortion	Ceftriaxone and Ampicillin (12 days), followed by only Ceftriaxone (2 days)	12 days
5.	Chai et al. [12]	2011	32	12	No	Unknown	Amenorrhea, fever, chills, vomiting	Fetal death followed by vacuum curettage	Meropenem	N/A
6.	Meirowitz et al. [11]	2006	28	21–25	No	Central catheter line	Fever, chills, malaise, headache	Emergency cesarean delivery	Ceftriaxone (11 days), followed by Ertapenem (17 days), followed by Imipenem (14 days)	41 days
7.	Shimizu et al. [10]	2003	26	10–20	Yes	Urinary tract	Gait disturbance, severe tenderness, flaring in the lower left extremity, fever	Spontaneous abortion	Cefotiam (5 days), followed by Ceftazidime (8 days), followed by Imipenem/Cilastatin (8 days). Second admission – Ceftriaxone and Imipenem/Cilastatin up to the abortion?, followed by Imipenem/Cilastatin (by the 7 day after the abortion 7)	40 days during first admission? Period of the second admission is N/A
8.	Prosser et al. [9]	2003	38	19–21	Yes	Chorionic-villus sampling	Intermittent fevers, malaise.	Spontaneous abortion	Tobramycin and Cefepime (12 days) followed by Trimethoprim–sulfamethoxazole and Cefepime (2 weeks?)	12 days

Potentially, two mechanisms of fetal loss are associated with infection, and both are characteristic of advanced pregnancy. First, bacterial invasion of the amniotic cavity or fetoplacental membranes can stimulate labor of an immature fetus, and second, intrauterine infection of the fetus can probably occur as a result of the swallowing or inhaling of infected amniotic fluid and cause fetal pneumonitis and/or septicemia [20].

FGR is defined as a condition in which the fetus does not achieve its genetically determined growth potential [23]. FGR can be caused by a variety of factors, such as infections, the mother’s illnesses, and chromosomal disorders, but it primarily refers to anomalies in placental development that occur early in pregnancy [24,25]. The pathophysiology of discordant FGR remains insufficiently and inaccurately defined. Several recognized factors, categorized as maternal, fetal, and placental, influence the likelihood of discordant twins [26]. Altogether, with factors including intrauterine surroundings and utero-placental insufficiency, this condition can lead to discordant FGR [26].

Discordant fetal growth (with greater than 20% discordance) complicates 15% to 29% of twin pregnancies [27]. In a study involving 15,066 twin pregnancies, the rate of miscarriage was significantly increased when discordance was greater than 20%, especially in cases of monochorionic twin pregnancies [28]. It is important to note that the pregnancy in this case report was dichorionic. The increased pregnancy loss rate is attributed to monochorionicity of twin pregnancy [29].

In this case, infection by *S. marcescens* cannot be excluded as a cause of discordant growth. This is supported by the lack of awareness of the pregnancy period when the infection occurred. Previous studies have shown that chronic chorioamnionitis in some cases may be the cause of restrictive intrauterine growth of the fetus, especially in twin pregnancies [30]. Thus, it is reported, on rare occasions, that bacterial infections such as chlamydia, mycoplasma, listeria, and tuberculosis can cause restrictive intrauterine growth [31]. In addition, studies are required to examine the association between localization and/or different bacterial populations with the degree of oxygenation and maternal and fetal circulation. The immunomodulatory effect caused by bacterial infections of the placenta, hypothetically, may be the reason for the restrictive growth of the fetus and its subsequent rejection or preterm birth, and this needs to be confirmed by further research.

## 4 Conclusions

Although rare, *S. marcescens* can cause spontaneous miscarriages and, in this case, loss of extremely preterm birth of discordant twins in an immunocompetent patient. Infection by *S. marcescens* cannot be excluded as a cause of discordant growth and needs to be confirmed by further research.

## Abbreviations

AC: abdominal circumference
BPD: biparietal diameter
CRP: C-reactive protein
DNA: deoxyribonucleic acid
EFW: estimated fetal weight
FGR: fetal growth restriction
FL: femur length
HC: head circumference
Hgb: hemoglobin
PCR: polymerase chain reaction
PLT: platelet count
RBCs: red blood cells
WBCs: white blood cells
WG: weeks of gestation
